# BISHOP–KOOP modification technique following proximal jejunal anastomosis: A case report

**DOI:** 10.1016/j.amsu.2020.06.027

**Published:** 2020-06-24

**Authors:** Vicky S. Budipramana, Putu Ayu Saraswati

**Affiliations:** Departement of Surgery, Dr. Soetomo Hospital Surabaya, Faculty of Medicine, Universitas Airlangga, Jl. Mayjen Prof. Dr. Moestopo 47, Surabaya, 60132, Indonesia

**Keywords:** Proximal jejunum, Bishop-koop anastomosis, Decompression, Nutrition tube, Case report

## Abstract

**Introduction:**

Very-short proximal jejunal stump anastomosis leak has been a major problem in surgery and it causes high postoperative morbidity and mortality. However, using a Bishop-Koop Modification technique anastomosis with decompression and nutrition tube, we can completely cure the patient with this case.

**Presentation of case:**

A 61-year-old man came to the emergency room with generalized peritonitis and sepsis, on emergency laparotomy we found a perforation from solid tumor located in the proximal jejunum, 20 cm distal to ligament of Treitz. Free purulent exudate and diffuse inflammatory reaction of the peritoneum were also found in the abdominal cavity. We resected the jejunum together with the mass and anastomosis using Bishop-Koop technique with the decompression and nutrition tube. The patient completely recovered and left the hospital after a total stay of 30 days.

**Discussion:**

Surgery-associated-anastomotic leak has been a major complication in performing anastomosis on the very-proximal jejunum especially in septic condition. The decompression after anastomosis is important, because of the high excretion of Brunner gland, bile, pancreas, duodenum, and jejunum juice and also the paralytic bowel condition in septic condition can make fluid accumulation in jejunum. It was impossible to decompress the anastomosis by performing an external jejunostomy because the proximal stump was too close to the ligament of Treitz. Using Bishop-Koop anastomosis technique, we were able to decompress the anastomosis and to give early nutrition using tubes at the same time.

**Conclusion:**

Bishop-Koop anastomosis modification with decompression and nutrition tube is a safe procedure for anastomosing on the very-proximal jejunum.

## Highlights

• Anastomotic leak has been the main reason for postoperative morbidity/mortality.
• It is difficult to perform anastomosis in proximal small bowel perforation.
• Bishop-Koop anastomosis technique was performed for the proximal jejunum.
• Modification of the technique was made to avoid anastomosis breakdown.
• 14 FR nasogastric tube & 14 FR catheter were used for decompression and nutrition.

## 1 Introduction

Gastrointestinal surgery-associated-anastomotic leak has been the major problem to postoperative morbidity and mortality [1]. The reports about the incidence rate of leak in a very-short proximal jejunal stump anastomosis have hardly ever been presented, it is contrary to about 1–3% rate of the anastomosis leak involving small intestine in general [1]. The risk of the anastomosis leak in proximal jejunal anastomosis is high because the decompression cannot be performed perfectly. The high excretion of Brunner gland, bile, pancreatic, duodenum and jejunum juice, and because the paralytic bowel condition after operation in septic condition make much fluid accumulation in jejunum which can lead to anastomosis breakdown [2]. The previous studies showed various gastrointestinal reconstructions to decompress the anastomosis such as gastrojejunostomy, Mikulicz double barrel ileostomy, Santulli reanastomosis with stapler, transstomal EndoVac™, and multiple drainage [[3], [4], [5], [6], [7], [8]], but the data are unexplored for these kinds of enterostomies. Those techniques need multiple enterotomies and multiple anastomosis which cause the higher risk of the anastomotic breakdown and also they need the next-step reconstruction surgeries. In this case, we decompressed the anastomosis more effectively by using Bishop-Koop technique with the decompression tube inserting from the distal limb of the jejunum together with the nutrition tube to provide early enteral nutrition. Moreover this technique requires only one-time surgery as the decompression and the nutrition tubes were easily taken out from the abdominal wall after the condition of the patient became stable [9]. Different from the previously reported studies which required more than one-time surgeries afterward. This work has been reported in line with the SCARE criteria [10].

## 2 Presentation of case

**Fig. 1:**
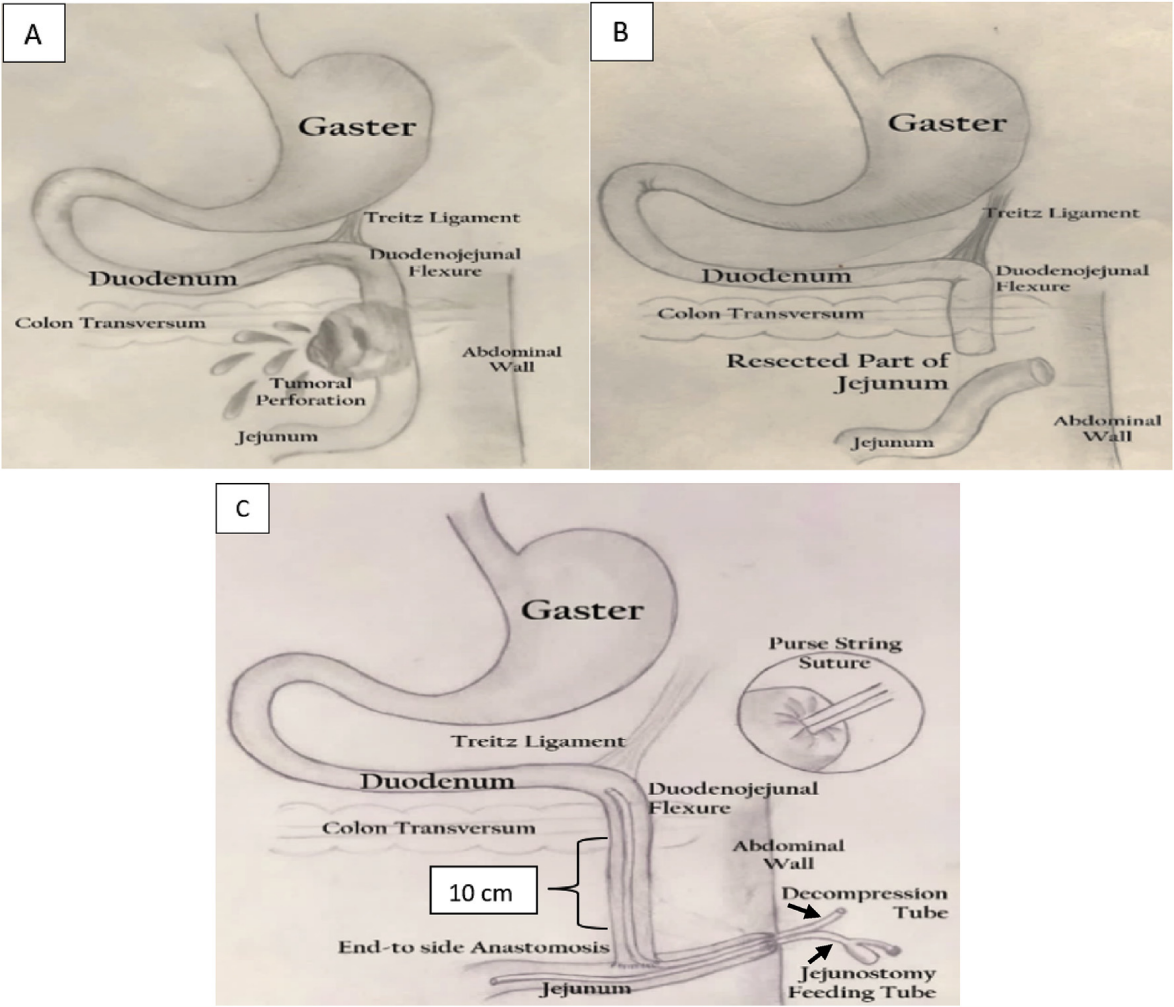
Operative diagram showing (A) Perforated GIST on proximal jejunum. (B) Short stump of proximal jejunum and the blind end of the distal part of jejunum after resection. (C) End-to-side anastomosis followed by decompression and nutrition tube (arrow).

A 61-year-old man was admitted to the emergency unit of our hospital with fever, vomiting, and diffuse abdominal pain that the patient had been suffering for the previous 24 hours. The patient had no history of allergies, drugs, and psychosocial disturbance. No other co-morbidities were found, the vital signs were **BP** 90/60 mmHg, HR 110 and the rectal temperature was 38.6 °C. On physical examination, we found out that the abdomen was distended with generalized tenderness and muscular rigidity. Laboratory tests showed elevated white blood cells (29.430/mm^3^, 87.4% neutrophils) and anemia (8.6 g/dL). The clinical diagnosis was perforation peritonitis from the unclear cause, the plain abdominal photo and CT Scan also could not show the source of perforation. We decided to perform an emergency laparotomy. On surgery, we found free purulent exudate in the abdominal cavity and diffuse inflammatory reaction of the peritoneum and we found out the leakage from the unsuspected 5 × 4 × 3 cm solid tumor located 20 cm distal from the ligament of Treitz on the antimesenteric site of jejunum. No metastatic nodes were found on the mesenterium, liver or other parts in the peritoneal cavity. We performed segmental resection of the jejunum 10 cm proximal and 10 cm distal from the outer margin of the tumor together with the tumor (Fig. 1a)**.** The direct end to side anastomosis of the jejunum was created, and the distal blind stump of the jejunum was brought out for decompression to protect the anastomosis. The decompression of the anastomosis by performing external jejunostomy was difficult because the length of the proximal stump of jejunum was not enough to bring it out as external stoma since the proximal jejunal stump was too close to the ligament of Treitz. Therefore we made an end-to-side manually sewn anastomosis using continuous monofilament technique at a point of 20 cm proximal from the blind end of the distal limb of jejunum, then the tip of distal limb was brought out through the abdominal wall as seen in (Fig. 1b). In addition to preventing anastomosis breakdown, we placed a 14 FR nasogastric tube upward through the anastomosis line for decompression and placed another 14 FR catheter downward for nutrition. These tubes were fixed to the parietal peritoneum with double purse-string using absorbable polyglactin 2-0 suture (Fig. 1c). We closed the abdomen after putting the drain into the abdominal cavity. The immunohistochemistry of the tumor in proximal jejunum confirmed the diagnosis of CD117-Positive GIST.

The patient was transferred to High Care Unit the 3rd day after surgery because of nosocomial pneumonia and sepsis which required intensive care nursing. The antibiotic Ceftriaxon 2 × 1 gr (IV) combined with Metronidaole 3 × 500 mg (IV) was given since the preoperative periode and changing to meropenem 3 × 1 gr (IV) + Metronidazole 3 × 500 mg (IV) when the patient was in the High Care Unit. The initial nutritional support was given through the central venous route. On the 4th day, there was no sign of anastomosis leakage, so we began to give him the enteral nutrition via distal catheter in order to establish the bowel function. The patient tolerated well with the enteral nutrition program. The output of decompression drain was between 200 and 300 ml per 24 hours from the day of operation up to the 15th day, and by the 20th day, the drain output was below 50 ml per 24 hours. The patient's condition progressed, the pneumonia subsided and moved to the ward after 5 days of treatment at the High Care Unit. By the 25th day the patient could eat normally, the decompression and the nutrition tubes were taken out, and the wound from the drains on the abdominal wall could be closed by itself. The patient left the hospital after a total stay of 30 days.

## 3 Discussion

Gastrointestinal surgery-associated anastomotic leak has been the major problem to postoperative morbidity and mortality [1]. Management of bowel perforation involves prompt surgical intervention, debridement of devitalized tissue, lavage, and primary anastomosis with or without partial resection, and anastomosis may be required depending on the location and the severity of perforation [11]. It is risky to perform anastomosis in proximal jejunum because of the high excretion of Brunner gland, duodenum and jejunum production, bile and pancreatic juice leading to subsequent anastomosis breakdown and also due to the septic condition caused by peritonitis and the paralytic bowel condition after operation can make fluid accumulation in jejunum leading to the anastomosis breakdown [2]. Various autologous gastrointestinal reconstructions such as gastrojejunostomy have been proposed in the literature to solve this problem. The procedure of gastrojejunostomy has been the most widely used one for obstructing or perforating lesions around the duodenojejunal flexure, but a long blind loop, stoma ulceration, and alkaline reflux gastritis are some of the major drawbacks of this procedure [3]. To prevent those drawbacks, we performed an end-to-side anastomosis between the end of the proximal jejunal stump and the side of distal jejunum. The distal limb of the jejunum was brought out as an external-stoma (Bishop-Koop stoma). We modified this technique by closing the distal limb of the jejunum and inserting 2 tubes for decompression and nutrition to protect the anastomosis from breakdown and to provide earlier nutrition (Fig. 1c).

Other procedures have been described such as Mikulicz double barrel ileostomy and the Santulli technique. The Santulli enterostomy is similar to the Bishop-Koop except that the proximal limb is brought out as the stoma and the distal limb for the end-to-side anastomosis. We did not use this technique because the length of the proximal part of jejunum was not enough to mobilize and for us to bring it out as external stoma. This type of Bishop-Koop modification anastomosis with decompression and nutrition tube in adult is not well described in the literature. However, one case using Bishop-Koop jejunostomy technique in proximal small bowel anastomosis to prevent high output proximal stoma has been reported [4]. The concern when directly repairing the area closed to duodenojejunal flexure injury is to protect the suture line from the proteolytic action of the large volume of the upper gastrointestinal secretion. If the anastomotic leakage occurs, the patients are at risk of either peritonitis or high enterocutaneous fistula. Another procedure such as re-anastomosis with stapler [5], transstomal EndoVac™ ^7^ [6], and multiple drainage [7,8] has been included in the studies comparing different methods for proximal bowel injuries to provide earlier introduction to oral intake and decrease the length of hospital stay, but the data have hardly ever been presented for these enterostomies.

The use of the tube for decompression has been proposed in the literature. Chen and Yang [12] emphasized the use of duodenostomy tube for protection of the duodenal suture line, and according to them, the incidence rate of fistula with or without the duodenostomy tube is 2.3% and 11.8% respectively. Another common technique is using ‘triple-tube’ decompression with nasogastric tube or gastrostomy, as retrograde and antegrade tubes for both duodenal decompression and jejunostomy feeding, respectively [7]. Crippa et al. [8] proposed a ‘quadruple tube’ decompression by using an additional T-tube in the common bile duct to reduce the possibility of the incidence of duodenal leakage. But the data about the success rate of these kinds of tube protections have hardly ever been presented.

In our case, simply placing the 14-Fr Foley catheter tube and another14-Fr NG tube through the anastomotic site helped to decompress the jejunal content without performing duodenostomy or gastrostomy. Moreover, we did not need to perform another surgery to close the distal end jejunostomy, as we just pulled out the decompression and nutrition tubes and let the wound tract on the abdominal wall closed by itself. This modified method protected the primary anastomosis and decreased the chance of duodenum-related complications.

## 4 Conclusion

Performing a direct end-to-end anastomosis in the proximal jejunal perforation in an infected abdomen case is risky for leakage because of the high jejunal content coupled with post-operative paralytic ileus due to the septic condition. The Bishop-Koop modification anastomosis technique by placing the decompression tube proximally and nutrition tube distally is a safe procedure for preventing anastomosis breakdown and providing earlier enteral nutrition.

## Ethical approval

Our case is exempt from ethical approval in our institute.

## Sources of funding

This research did not receive any specific grant from funding agencies in the public, commercial, or not-for-profit sectors.

## Author contribution

**Vicky S. Budipramana:** study concept and design, paper writing and editing.

**Putu Ayu Saraswati:** data collection.

## Trial registry number

This manuscript is a case report, not a research study.

## Guarantor

The guarantor of this study is Vicky S. Budipramana.

## Consent of patient

Written informed consent was obtained from the patient for publication of this case report and accompanying images.

## Provenance and peer review

Not commissioned, externally peer reviewed.

## Declaration of competing interest

None.
